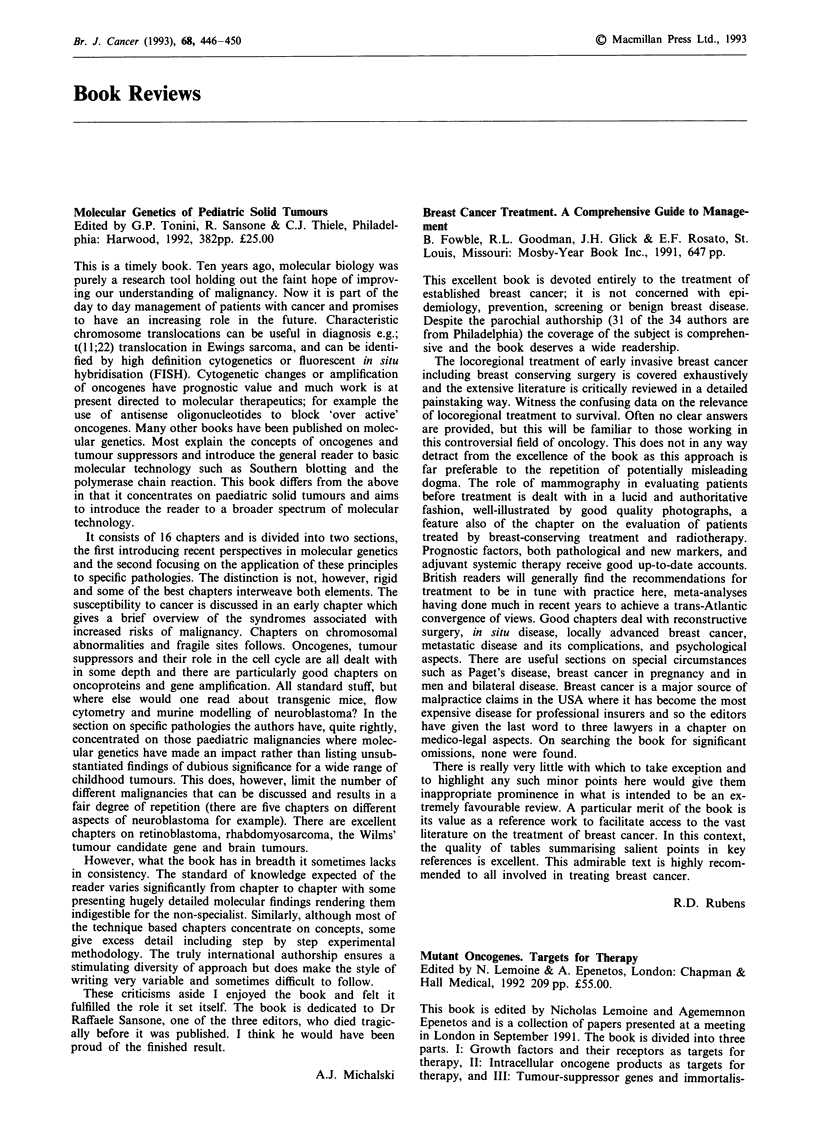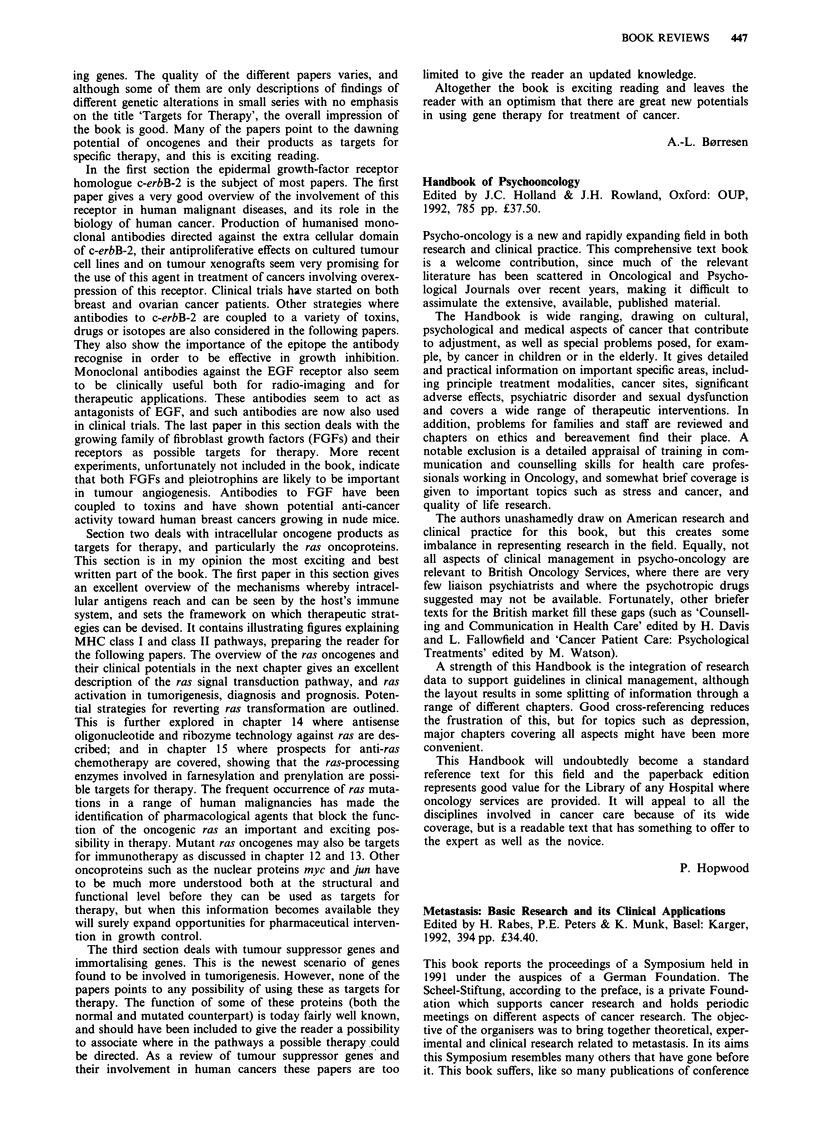# Mutant Oncogenes. Targets for Therapy

**Published:** 1993-08

**Authors:** A.-L. Børresen


**Mutant Oncogenes. Targets for Therapy**
Edited by N. Lemoine & A. Epenetos, London: Chapman & Hall Medical, 1992 209 pp. £55.00.

This book is edited by Nicholas Lemoine and Agememnon Epenetos and is a collection of papers presented at a meeting in London in September 1991. The book is divided into three parts. I: Growth factors and their receptors as targets for therapy, II: Intracellular oncogene products as targets for therapy, and III: Tumour-suppressor genes and immortalising genes. The quality of the different papers varies, and although some of them are only descriptions of findings of different genetic alterations in small series with no emphasis on the title 'Targets for Therapy', the overall impression of the book is good. Many of the papers point to the dawning potential of oncogenes and their products as targets for specific therapy, and this is exciting reading.

In the first section the epidermal growth-factor receptor homologue c-*erb*B-2 is the subject of most papers. The first paper gives a very good overview of the involvement of this receptor in human malignant diseases, and its role in the biology of human cancer. Production of humanised monoclonal antibodies directed against the extra cellular domain of c-*erb*B-2, their antiproliferative effects on cultured tumour cell lines and on tumour xenografts seem very promising for the use of this agent in treatment of cancers involving overexpression of this receptor. Clinical trials have started on both breast and ovarian cancer patients. Other strategies where antibodies to c-*erb*B-2 are coupled to a variety of toxins, drugs or isotopes are also considered in the following papers. They also show the importance of the epitope the antibody recognise in order to be effective in growth inhibition. Monoclonal antibodies against the EGF receptor also seem to be clinically useful both for radio-imaging and for therapeutic applications. These antibodies seem to act as antagonists of EGF, and such antibodies are now also used in clinical trials. The last paper in this section deals with the growing family of fibroblast growth factors (FGFs) and their receptors as possible targets for therapy. More recent experiments, unfortunately not included in the book, indicate that both FGFs and pleiotrophins are likely to be important in tumour angiogenesis. Antibodies to FGF have been coupled to toxins and have shown potential anti-cancer activity toward human breast cancers growing in nude mice.

Section two deals with intracellular oncogene products as targets for therapy, and particularly the *ras* oncoproteins. This section is in my opinion the most exciting and best written part of the book. The first paper in this section gives an excellent overview of the mechanisms whereby intracellular antigens reach and can be seen by the host's immune system, and sets the framework on which therapeutic strategies can be devised. It contains illustrating figures explaining MHC class I and class II pathways, preparing the reader for the following papers. The overview of the *ras* oncogenes and their clinical potentials in the next chapter gives an excellent description of the *ras* signal transduction pathway, and *ras* activation in tumorigenesis, diagnosis and prognosis. Potential strategies for reverting *ras* transformation are outlined. This is further explored in chapter 14 where antisense oligonucleotide and ribozyme technology against *ras* are described; and in chapter 15 where prospects for anti-*ras* chemotherapy are covered, showing that the *ras*-processing enzymes involved in farnesylation and prenylation are possible targets for therapy. The frequent occurrence of *ras* mutations in a range of human malignancies has made the identification of pharmacological agents that block the function of the oncogenic *ras* an important and exciting possibility in therapy. Mutant *ras* oncogenes may also be targets for immunotherapy as discussed in chapter 12 and 13. Other oncoproteins such as the nuclear proteins *myc* and *jun* have to be much more understood both at the structural and functional level before they can be used as targets for therapy, but when this information becomes available they will surely expand opportunities for pharmaceutical intervention in growth control.

The third section deals with tumour suppressor genes and immortalising genes. This is the newest scenario of genes found to be involved in tumorigenesis. However, none of the papers points to any possibility of using these as targets for therapy. The function of some of these proteins (both the normal and mutated counterpart) is today fairly well known, and should have been included to give the reader a possibility to associate where in the pathways a possible therapy could be directed. As a review of tumour suppressor genes and their involvement in human cancers these papers are too limited to give the reader an updated knowledge.

Altogether the book is exciting reading and leaves the reader with an optimism that there are great new potentials in using gene therapy for treatment of cancer.

A.-L. Børresen